# Multi-Marker Approach for the Identification of Different *Heterodera* Species (Nematoda: Heteroderidae)

**DOI:** 10.3390/pathogens14101052

**Published:** 2025-10-18

**Authors:** Maria João Camacho, Maria L. Inácio, Eugénia de Andrade

**Affiliations:** 1 INIAV—National Institute for Agriculture and Veterinary Research, Quinta do Marquês, 2780-159 Oeiras, Portugal; mjoao.camacho@iniav.pt (M.J.C.); eugenia.andrade@iniav.pt (E.d.A.); 2 GREEN-IT Bioresources for Sustainability, ITQB NOVA, Av. da República, 2780-157 Oeiras, Portugal

**Keywords:** *Heterodera schachtii*, *Heterodera cruciferae*, *Heterodera trifolii*, *Heterodera mani*, *Heterodera zeae*, mt*COI*, 18S, ITS, 28S rDNA

## Abstract

Cyst nematodes of the genus *Heterodera* are important plant-parasitic nematodes that cause significant crop losses worldwide but are often overlooked due to their non-specific symptoms and complex biology. This study assessed *Heterodera* diversity in Portugal using an integrative molecular approach based on four genetic markers (mt*COI*, 18S rDNA, ITS, and 28S rDNA). Five valid species were identified: *Heterodera cruciferae*, *H. mani*, *H. schachtii*, *H. trifolii*, and *H. zeae*, with *H. mani* reported for the first time in the country. A distinct taxon from Coimbra (central Portugal) may represent a new or unsequenced species, highlighting gaps in reference datasets. Among the markers, mt*COI* was the most effective, though some taxa remained unresolved. These results reinforce the value of multi-marker approaches, contribute with new sequences, and improve diagnostic capability for nematode management.

## 1. Introduction

Nematodes of the genus *Heterodera*, commonly known as cyst nematodes, are an ancient group of plant-parasitic nematodes (PPNs) that seriously constrain crop production worldwide [1]. Over 80 species of *Heterodera* have been described [2], and their management is difficult due to the fact that cysts can remain dormant in soil for long periods, with juveniles hatching only under favourable conditions [3]. Despite their impact, they are often overlooked, as symptoms are easily mistaken for abiotic stress [4].

Generally, this genus can be easily recognized by the lemon shaped cysts formed from the dead female body, holding hundreds of viable eggs, while species identification is challenging, requiring specialized expertise and often leading to misidentifications [5]. This is due to the variability in morphological characteristics and morphometric values within the same species and their overlap among species [2], compromising accurate identification and often resulting in misidentifications. Additionally, few species cannot be distinguished based on morphology alone. Given their agronomic importance, accurate species identification is needed not only to implement effective measures for *Heterodera* spp. management but also to address regulatory concerns associated with certain species. For instance, *Heterodera glycines* Ichinohe, 1952, is listed as a harmful quarantine organism on the European and Mediterranean Plant Protection Organization (EPPO) A2 List (A2/167) [6]. Prompt and accurate identification of such species is critical to ensure compliance with quarantine regulations and to support the development of effective control measures, thereby safeguarding both regional and international agricultural practices.

Molecular tools have therefore become crucial for species diagnosis [1]. Early approaches relied on PCR and PCR-RFLP, but sequencing now predominates, with over 40 species represented in GenBank [5] Commonly used markers include mt*COI*, 18S rDNA, ITS, and 28S rDNA [7,8,9,10,11,12,13,14,15]. However, identical ITS sequences are shared among morphologically distinct species (e.g.,: *H. avenae* Wollenweber, 1924/*H. arenaria* Cooper, 1955; *H. carotae* Jones, 1950/*H. cruciferae* Franklin, 1945; or *H. trifolii* Goffart, 1932/*H. daverti* Wouts & Sturhan, 1978) [8]. These pairs of species cannot be reliably distinguished using one or more commonly targeted genes [1,5,8,9,12,15,16,17]. For instance, *H. avenae* Wollenweber, 1924 and *H. pratensis* Gäbler et al., 2000 share identical ITS and 28S rDNA sequences. Similarly, identical 18S rDNA and ITS regions are shared among *H. schachtii* Schmidt, 1871, *H. betae* Wouts, et al., 2001, and *H. trifolii* Goffart, 1932. In the 28S rDNA region, *H. schachtii* differs from *H. betae* by only one base pair (1 bp) and from *H. trifolii* by just two base pairs [1]. Among markers, mt*COI* provides the greatest discriminatory power and should be favoured over 18S, ITS, and 28S markers when resources are limited [1]. In conclusion, intraspecific polymorphism and sequence similarities among species can complicate accurate identification, highlighting the continued need for more conclusive and robust molecular tools [5]. Nonetheless, reliance on molecular data also presents its own challenges. Public databases such as GenBank and BOLD contain numerous sequences that are incomplete, of low quality, or potentially misidentified—often due to the lack of rigorous morphological validation or taxonomic oversight. This problem is particularly relevant for underrepresented or cryptic species within the genus *Heterodera*, where high intraspecific variation or interspecific similarity can lead to ambiguous results.

Currently, in Portugal, there is a lack of detailed information on *Heterodera* spp. presence. According to Reis [18], the first occurrence of cyst nematodes in Portugal was *H. schachtii*, identified by Oliveira in 1943 [19]. Later, Macara [20,21,22] reported the occurrence of *H. goettingiana* Liebscher, 1892 on peas and broad beans, *H. avenae* on wheat, *H. cruciferae* on cabbage, and *H. fici* Kirjanova, 1954 associated with fig trees. Posteriorly, Correia [23] reported the occurrence of nine *Heterodera* species in Portugal—*H. avenae*, *H. carotae*, *H. cruciferae*, *H. daverti*, *H. fici*, *H. goettingiana*, *H. schachtii*, *H. urticae* Cooper, 1955, and *H. zeae* Koshy, Swarup & Sethi, 1971. Madani et al. [24] referred Portuguese isolates of *H. fici* and *H. humuli* Filip’ev, 1934 in his study of molecular characterization of cyst nematodes from the Mediterranean basin. *Heterodera hordecalis* Andersson, 1975 and *H. trifolii* were mentioned in the list of the terrestrial nematodes from Azores [25]. Finally, Gracianne et al. [26] showed that *H. betae* is widely distributed along the Portuguese Atlantic coastline, associated with sea beet (*Beta vulgaris* ssp. *maritima*), a wild beet relative. Amongst others, as far as is known, *H. mani* Mathews, 1971 has never been reported in Portugal.

In this study, we investigated *Heterodera* diversity in Portugal exclusively through molecular markers, addressing the absence of reliable morphological data. We sequenced four regions (mt*COI*, 18S rDNA, ITS, and 28S rDNA), evaluated sequence quality, and validated database matches. Our objectives were to (i) identify the *Heterodera* species present, (ii) provide high-quality sequences to public databases, and (iii) assess the strengths and limitations of marker-based identification. By achieving this, we contribute to improved diagnostic tools, more accurate monitoring of cyst nematodes, and better support for management and regulatory frameworks in agriculture.

## 2. Materials and Methods

### 2.1. Sampling

Soil samples were collected between 2018 and 2023 from various regions across Portugal during the official survey for potato cyst nematodes (*Globodera* spp.) from the National Phytosanitary Authority (DGAV, Lisboa, Portugal). Sampling was carried out in potato fields after harvest according to Annex II of DL 87/2010, and soil samples were stored in plastic bags and coded, ensuring the anonymity of the samples during the analysis period.

### 2.2. Heterodera spp. Isolates

Cysts of nematodes were extracted from soil samples following the EPPO PM 7/119 (1) and PM 7/40 (5) protocols [27,28] using the Fenwick’s can method [29], and *Heterodera* cysts were subsequently isolated. Viable juveniles from selected cysts were entirely reserved for DNA extraction, making morphological and morphometric analyses not possible within the scope of this study. Given the focus on molecular diagnostics, priority was given to obtaining high-quality DNA.

### 2.3. DNA Purification and Amplification

DNA was independently extracted from the juveniles present in one viable cyst per isolate using a QIAamp^®^ DNA Mini Kit (Qiagen, Hilden, Germany) following the manufacturer’s instructions. All DNA samples were stored at −20 °C until further use. Given the anticipated low DNA concentration, both the concentration and the quality were estimated by spectrophotometry.

The mt*COI* gene region was amplified using the Hu et al. primers [30] JB3 (5′-TTT TTT GGG CAT CCT GAG GTT TAT-3′) and JB5 (5′-AGC ACC TAA ACT TAA AAC ATA ATG AAA ATG-3′). The expected length of PCR fragments was 447 bp. The thermal cycling conditions performed consisted of an initial denaturation of 98 °C for 1 min followed by 40 cycles of 98 °C for 10 s, 41 °C for 20 s, and 72 °C for 30 s, and a final extension of 72 °C for 10 min.

PCR reactions were performed in a 20 μL final volume containing 5 μL of template DNA, 12.5 μL of Supreme NZYTaq II 2× Green Master Mix (NZYTech, Lisbon, Portugal), 1.5 μL of each primer (10 μM), and 4.5 μL of water in a Biometra TGradient thermocycler (Biometra, Gottingen, Germany).

The 18S gene of rDNA was amplified using primers described by Holterman et al. [31] in two overlapping fragments—988F/1912R and 1813F/2646R (988F: 5′-CTC AAA GAT TAA GCC ATG C-3′, 1912R: 5′-TTT ACG GTC AGA ACT AGG G-3′, 1813F: 5′-CTG CGT GAG AGG TGA AAT-3′ and 2646R: 5′-GCT ACC TTG TTA CGA CTT TT-3′). The expected length of PCR fragments was approximately 980 and 880 bp, respectively, resulting in an approximately 1730 bp amplicon size including primers [32].

The thermal cycling conditions performed consisted of an initial denaturation of 94 °C for 5 min followed by 53 cycles of 94 °C for 30 s, 45 °C for 30 s, and 72 °C for 70 s, followed by 35 cycles of 94 °C for 30 s, 54 °C for 30 s, and 72 °C for 70 s, and a final extension of 72 °C for 10 min. PCR reactions were performed in a 25 μL final volume containing 5 μL of template DNA, 12.5 μL of Supreme NZYTaq II 2× Green Master Mix (NZYTech, Lisbon, Portugal), 1.5 μL of each primer (10 μM), and 4.5 μL of water in a Biometra TOne Gradient thermocycler (Biometra, Gottingen, Germany).

The ITS-rDNA region was amplified using primers developed by Ferris et al. [14]: 18L (5′-CGT AAC AAG GTA GCT GTA G-3′) and ITS4mod (5′-TCC TCC GCT AAA TGA TAT G-3′). The expected length of PCR fragments was 1040 bp and encompassed the 3′ end of 18S rDNA–ITS1–5.8S–ITS2 and the 5′ end of the 28S rDNA. The thermal cycling conditions performed consisted of an initial denaturation of 95 °C for 5 min followed by 40 cycles of 94 °C for 30 s, 55 °C for 30 s, and 72 °C for 33 s, and a final extension of 72 °C for 7 min. PCR reactions were performed in a 25 μL final volume containing 3 μL of template DNA, 12.5 μL of Supreme NZYTaq II 2× Green Master Mix (NZYTech, Lisbon, Portugal), 0.6 μL of each primer (10 μM), and 8.3 μL of water in a Biometra TOne Gradient thermocycler (Biometra, Gottingen, Germany).

The 28S rDNA region was amplified using the De Ley et al. primers [33] D2A (5′-ACA AGT ACC GTG AGG GAA AGT TG-3′) and D3B (5′-TCG GAA GGA ACC AGC TAC TA-3′). The expected length of PCR fragments was 780 bp. The thermal cycling conditions performed consisted of an initial denaturation of 95 °C for 10 min followed by 40 cycles of 95 °C for 30 s, 60 °C for 45 s, and 72 °C for 45 s, and a final extension of 72 °C for 10 min. PCR reactions were performed in a 25 μL final volume containing 2 μL of template DNA, 12.5 μL of Supreme NZYTaq II 2× Green Master Mix (NZYTech, Lisbon, Portugal), 0.75 μL of each primer (10 μM), and 9 μL of water in a Biometra TOne Gradient thermocycler (Biometra, Gottingen, Germany).

### 2.4. Sequencing

Amplified products were loaded onto a 1.5% agarose gel in TAE and subjected to electrophoresis at 5 V/cm in a Mupid One System (Nippon Genetics Europe, Düren, Germany). This system allows for the visualization and detection of DNA fragments during the run using direct staining of DNA with Midori Green (Nippon Genetics Europe, Düren, Germany) together with safe blue LEDs that do not degrade or mutate DNA. Possible contaminations were checked by including negative controls (no template control—NTC) in all amplifications.

In all situations, the expected single amplicons were visualized. The PCR products were enzymatically purified using ExoSAP-IT PCR Product Cleanup (Thermo Fisher, Waltham, MA, USA) following the manufacturer’s instructions, with incubation for 15 min at 37 °C followed by 15 min at 85 °C.

Cycle sequencing was performed with an ABI BigDye Cycle sequencing kit (Applied Biosystems, Carlsbad, CA, USA) on an ABI Prism 3130XL capillary sequencer in both directions using the same primers as for the PCR.

Sanger sequencing was outsourced at StabVida (Caparica, Portugal) and at another molecular biology laboratory of INIAV (Oeiras, Portugal).

### 2.5. Heterodera spp. Phylogenetic Analysis

The chromatograms generated during the sequencing analysis were visualized, and nucleotide sequences were edited and analyzed using BioEdit v7.2.0 (Ibis Biosciences, Carlsbad, CA, USA) [34], Geneious Prime version 2022.2.1 (Auckland, New Zealand) [35], and MEGA X version 10.2.6 (Pennsylvania State University, University Park, PA, USA) [36]. Unidirectional sequences were considered successful when the sequence of the complementary primer sequence was present at the 3′end, no double peaks were observed, and high fluorescence signals were detected along the entire sequence. Moreover, the mt*COI* sequences were translated using the invertebrate mitochondrial genetic code and aligned. In any of the sequences, no premature stop codons were detected. Once all quality criteria were fulfilled, primer sequences were trimmed, and consensus sequences were generated from the forward and reverse sequences.

The resulting consensus sequences for mt*COI*, 18S rDNA, ITS, and 28S rDNA were used as queries in BLAST + (v2.15.0; NCBI, 2023) searches [37] against the NCBI GenBank database to retrieve the most similar sequences among *Heterodera* species. All consensus sequences were subsequently deposited in the GenBank database (NCBI). Sequence alignments were checked by visual inspection.

### 2.6. Phylogenetic Analysis

As an initial step in the phylogenetic analyses, the aligned sequences were used to construct a distance tree using the Neighbor-Joining (NJ) method available at the NCBI platform. Although not the most robust approach, this method is widely used in DNA barcoding studies due to its simplicity and effectiveness in preliminary analysis.

Subsequently, all sequences were aligned using CLUSTALW [38] with default parameters. The primers regions were manually trimmed, and the pairwise-aligned sequences were further phylogenetically analyzed by the Maximum Likelihood method and the Tamura–Nei Genetic Distance model [39] implemented in MEGA X version 10.2.6 (Pennsylvania State University, University Park, PA, USA) [36].

A bootstrap analysis with 1000 replications was used to infer robustness of the resulting phylogenetic trees. Sequences from *G. rostochiensis* were used as an outgroup taxon.

## 3. Results

The nucleotide sequences generated in this study were deposited in the GenBank database of the National Centre of Biotechnology Information (NCBI) under the accession numbers given in Table 1.

Table 2 provides the nucleotide sequences of *Heterodera* spp. available from GenBank and used for the phylogenetic analysis, and Table 3 presents a sequence comparison of isolates with database references and resulting species identification.

The isolate from Coimbra (PQ462047, PQ686664, PQ621793, and PQ686673) consistently formed a distinct sub-clade across multiple markers, suggesting a potentially undescribed taxon or a species not yet represented in public databases. However, without morphological and morphometric data, its taxonomic position remains unresolved, and it is provisionally reported here as *Heterodera* sp.

### 3.1. Partial mtCOI Gene Sequence

Primers JB3/JB5 [30] amplified a DNA fragment of 447 bp in all isolates. After trimming the primer regions, a shorter fragment of 390 bp—characterized by high sequence variability—was retained and used for phylogenetic analysis.

**Figure 1:**
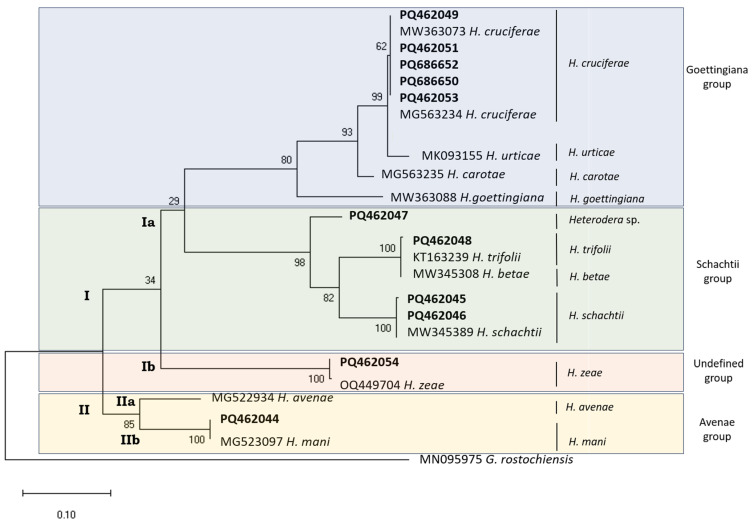
Phylogenetic relationships of *Heterodera* isolates from Portugal (bold) inferred from mt*COI* gene region. The evolutionary history was reconstructed by using the Maximum Likelihood method with the Tamura–Nei model [39] in MEGA X [36]. Bootstrap support (1000 replicates) indicated next to branches. Initial trees for the heuristic search were generated via Maximum Parsimony. Rate variation modeled with discrete Gamma (+G, 5 categories, 0.6167) with 31.55% invariable sites (+I). Branch lengths represent substitutions per site. Dataset: 23 sequences, 390 positions. *Globodera rostochiensis* used as outgroup taxon.

The phylogenetic relationships of *Heterodera* species based on the mt*COI* gene region (Figure 1) show two major clades: The first clade (I) is composed of two sub-clades corresponding (Ia) to the Goettingiana group (in blue) and the Schachtii group (in green) and (Ib) to the *H. zeae* (in orange) sequences (PQ462054 and OQ449704), allowing for the identification of the isolate from Santarém/Golegã (PQ462054) as *H. zeae.* The second clade (II) is the *Avenae* group clade (in yellow) and contains two sub-clades—one (IIa) with *H. mani* sequences (PQ462044 and MG523097) and another (IIb) with *H. avenae* sequence (MG522934).

Within the *Schachtii* group clade (in green), two sub-clades are evident. The first includes a single unidentified species sequence (PQ462047) from Coimbra, located in central Portugal. The second is divided between one group with *H. schachtii* sequences (MW345389, PQ462045, and PQ462046) and another group that contains *H. trifolii* sequences (PQ462048 and KT163239) and *H. betae* (MW345389), all grouped within the same clade.

In the Goettingiana group clade (in blue), sequences of *H. goettingiana* (MW363088), *H. carotae* (MG563235), and *H. urticae* (MK093155) form distinct sub-clades. In contrast, sequences of *H. cruciferae* (MW363073, PQ462049-PQ462053, and MG563234) are clustered together within the same sub-clade. All tested sequences showed over 99.74% similarity with homologous sequences deposited in NCBI database (Table 3), allowing for reliable species identification (Figure 1). Despite forming a single sub-cluster, isolates from Porto, Lisbon, Aveiro, Santarém/Salvaterra, and Vila Real (PQ462049-PQ462053) showed 100% similarity (100% of cover) with *H. cruciferae* sequences and 99.71% similarity (87% of cover) with *H. urticae* sequence (based on the only available *H. urticae* sequence, MK093155). These results support their identification as *H. cruciferae*, although confirmation using additional genetic markers is recommended.

The isolate from Viana do Castelo (PQ462048) showed 99.74% similarity with both *H. trifolii* and *H. betae* sequences (Table 3), differing by only one base pair from each. This level of similarity highlights the need for further comparison using additional genetic markers to confirm its species identify.

The sequence PQ462047, obtained from Coimbra, shares less than 93% similarity with any *Heterodera* species sequences currently available in the NCBI database (Table 3). In the pairwise comparisons tree of *Heterodera* sequences (Supplementary Figure S1), PQ462047 forms a unique sub-clade.

Notably, the BOLD Identification System (IDS) for the mt*COI* gene could not be used to analyze sequences obtained with primers JB3/JB5, as the expected length of PCR fragments was 447 bp, shorter than the minimum 500 bp required for species-level identification in BOLD.

### 3.2. ITS Region

The ITS region, in its broadest range, was analyzed using three primer pairs covering the 18S, ITS1, 5.8S, ITS2, and 28S segments. While all isolates successfully amplified the expected fragment, sequencing did not always yield complete coverage of the entire fragment for all the isolates.

#### 3.2.1. 18S rDNA

PCR amplification with primers 988F/1912R and 1813F/2646R [31] successfully generated the expected fragments, but sequencing was not fully successful for the isolates collected in Lisbon (PQ686667) and Porto (PQ686666), yielding sequences shorter than expected (Table 1). Additionally, no amplification was obtained for the isolate from Vila Real.

**Figure 2:**
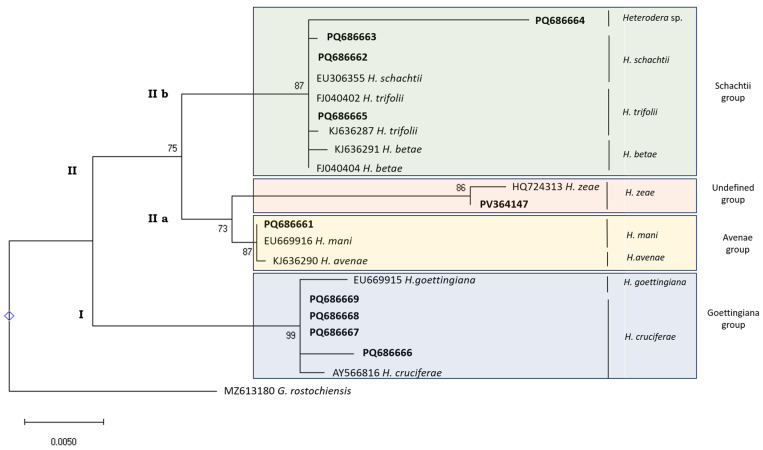
Phylogenetic relationships of *Heterodera* isolates collected from Portugal (bold) inferred from 18S rDNA region. The evolutionary history was reconstructed by using the Maximum Likelihood method with the Tamura–Nei model [39] in MEGA X [36]. Bootstrap support (1000 replicates) indicated next to branches. Initial trees for the heuristic search were generated via Maximum Parsimony. Rate variation modeled with discrete Gamma (+G, 5 categories, 0.0500) and 47.96% invariable sites (+I). Branch lengths represent substitutions per site. Dataset: 21 sequences, 1704 positions. *Globodera rostochiensis* used as outgroup taxon.

The phylogenetic relationships of *Heterodera* species based on the 18S rDNA region (Figure 2) showed two major clades. One clade (I) corresponds to the *Goettingiana* group (in blue), including sequences from *H. cruciferae* and *H. goettingiana*. In the other clade (II), two sub-clades are formed: one (IIa) contains the undefined group (in orange) with sequences of *H. zeae* (HQ724313 and PV364147), and the *Avenae* group (in yellow), clustering *H. mani* (PQ686661 and EU669916) and *H. avenae* (KJ636290); finally, the other sub-clade (IIb), is the *Schachtii* group (in green) which includes the unidentified species from Coimbra (PQ686664), *H. betae* (FJ040404 and KJ636291), *H. schachtii* (EU306355, PQ686662 and PQ686663), and *H. trifolii* (FJ040402 and PQ686665).

**Figure 3:**
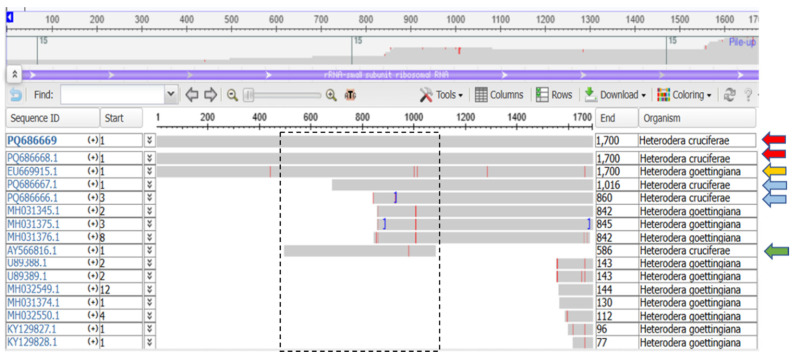
Comparison of *Heterodera goettingiana* and *H. cruciferae* sequences from NCBI. Red arrows: *H. cruciferae* sequences (Santarém PQ686669, Aveiro PQ686668); yellow arrow: *H. goettingiana* sequence (EU669915); blue arrows: partial *H. cruciferae* sequences (Porto PQ686666, Lisbon PQ686667); green arrow: partial *H. cruciferae* sequence (AY566816). Black dashed box: regions of overlap/non-overlap among aligned sequences.

Prior to this study, only a single *H. cruciferae* (AY566816) sequence based on the 18S rDNA region was available in the NCBI database (green arrow in Figure 3). This sequence, spanning only 586 bp (from 500 bp to 1085 bp of the expected approximately 1700 bp fragment) limited its effectiveness for accurate species identification (black dashed box in Figure 3). When combined with the truncated sequences obtained for the isolates from Porto (PQ686666) and Lisbon (PQ686667) (blue arrows in Figure 3)—which span from nucleotide position 860 and 1016 to the fragments’ end, respectively—there was an increased risk of misidentification of these isolates (Figure 3).

The issue lies in the length of the sequences and the overlap region. The sequences from Aveiro (PQ686668) and Santarém (PQ686669) (red arrows in Figure 3) are complete and show high similarity to *H. goettingiana* (EU669915—yellow arrow in Figure 3). The ones from Porto (PQ686666) and Lisbon (PQ686667) have only 245 bp of overlap with *H. cruciferae* (green arrow in Figure 3) but 875 bp with *H*. *goettingiana*. These 245 bp are identical between the two species. Therefore, based on this information alone, it would not be possible to distinguish between the two species. However, taking our knowledge based on the mt*COI* results which identified sequences of Porto and Lisbon as *H. cruciferae*, we can conclude that we are contributing with *H. cruciferae* sequences (PQ686666–PQ686669) that are distinguishable from *H. goettingiana* (Figure 3).

Indeed, the sequences from the isolates from Porto (PQ686666), Lisbon (PQ686667), Aveiro (PQ686668), and Santarém/Salvaterra (PQ686669) were all identified as *H. goettingiana* species (over 99% similarity—Table 3). However, sequences from the Santarém/Salvaterra (PQ686669), Aveiro (PQ686668), Porto (PQ686666), and Lisbon (PQ686667) isolates clustered with *H. cruciferae* and *H. goettingiana* sequences when the NCBI tree of pairwise comparisons of *Heterodera* sequences was generated (Supplementary Figure S2), supporting its fragile identification as *H. cruciferae*. The isolate species identification as *H. cruciferae* is supported based on the mt*COI* gene region, which clearly differentiates *H. cruciferae* sequences from the *H. goettingiana* sequence. There are no available *H. carotae* and *H. urticae* 18S gene sequences in the NCBI database for comparison.

A single sequence of *H. mani* (EU669916) is available in the NCBI database, based on the 18S rDNA region, but it was sufficient to identify the isolate from Castelo Branco (PQ686661) as *H. mani*, with 99.9% similarity (Table 3). Four sequences of *H. trifolii* and two of *H. betae* are available at NCBI database and all have high similarity (above 99.8%—Table 3) with the sequence of the isolate from the Viana do Castelo (PQ686665), as these sequences differ by one or two nucleotides. Despite the fact that the *H. trifolii* species sequences are clustered, the bootstrap value is not strong enough to identify the PQ686665 species as *H. trifolii*. A confirmation through other gene markers is still needed to identify this isolate species. These results are in line with other studies, which reported that ITS gene regions are shared between *H. schachtii*, *H. betae*, and *H. trifolii* [1].

The sequence from the isolate from Coimbra (PQ686664) has 99.72% similarity with EU306355 (*H. schachtii*) and FJ040404 (*H. betae*) and 99.65% similarity with KJ636291 (*H. betae*), KJ934138 (*H. glycines*), and KJ636284 (*H. schachtii*) (Table 3). However, when the NCBI tree of *Heterodera* sequences is generated using pairwise comparisons (Supplementary Figure S3), PQ686664 clusters separately rather than grouping with the sequences of *H. schachtii*, *H. betae*, or *H. glycines*.

#### 3.2.2. ITS-rDNA (18S rDNA–28S rDNA)

With the primer pair 18L/ITS4mod [14], all isolates except those collected in the region of Porto, Vila Real, and Santarém/Salvaterra amplified the expected fragment. After the trimming of the primers, sequences varying between 961 bp and 984 bp were used for the phylogenetic analysis.

**Figure 4:**
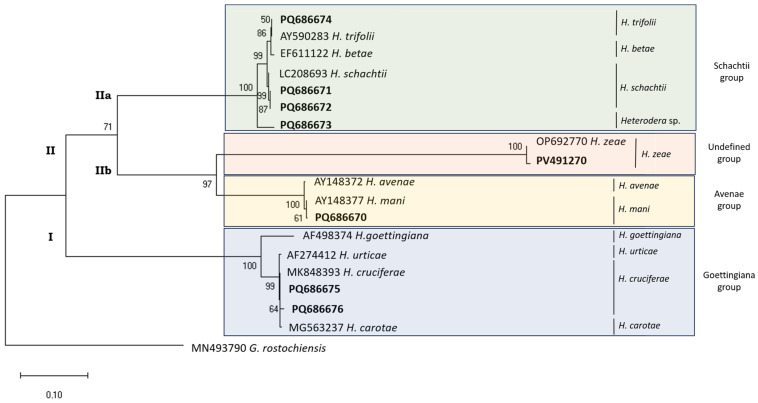
Phylogenetic relationships of *Heterodera* isolates from Portugal (bold) inferred from ITS-rDNA sequences. The evolutionary history was reconstructed using the Maximum Likelihood method with the Tamura–Nei model [39] in MEGA X [36]. Bootstrap support (1000 replicates indicated next to branches. Initial trees for the heuristic search were generated via Maximum Parsimony. Rate variation modeled with discrete Gamma (+G, 5 categories, 3.2494) with 27.41% invariable sites (+I). Branch lengths represent substitutions per site. Dataset: 19 sequences, 1000 positions. *Globodera rostochiensis* used as the outgroup taxon.

The phylogenetic relationships of *Heterodera* species based on the ITS-rDNA region (Figure 4) shows two major clades. Clade I corresponds to the Goettingiana group (in blue) and comprises *H. cruciferae* (MK848393, PQ686675, and PQ686676), *H. carotae* (MG563237), *H. urticae* (AF274412), and *H. goettingiana* (AF498374) species sequences. In clade II, two sub-clades are formed: the first sub-clade (IIa) (Schachtii group—in green) gathers *H. schachtii* (LC208693, PQ686671, and PQ686672), *H. betae* (EF611122), and *H. trifolii* (AY590283) species sequences and the unidentified species from Coimbra (PQ686673). The second sub-clade (IIb) is divided between the Avenae group (in yellow) that clusters *H. avenae* (AY148372) and *H. mani* (AY148377 and PQ686670) sequences and the undefined group with *H. zeae* sequences (OP69270 and PV491270). The sequences in this gene region are highly variable, allowing for the identification of isolates from Leiria (PQ686671) and Faro (PQ686672) as *H. schachtii*, highly supported by a 99% bootstrap value (Figure 4); the isolate from Viana do Castelo (PQ686674) as *H. trifolii* (100% similarity—Table 3), clearly distinguishing it from *H. betae* (99.27% similarity, with 7 different nucleotides); and the isolates from Lisbon (PQ686675) and Aveiro (PQ686676) as *H. cruciferae* (100% similarity), effectively ruling out misidentification with *H. urticae* or *H. carotae* (Table 3).

This marker alone could not to identify the isolate from Castelo Branco (PQ686670) as *H. mani*. Its identification is based on the mt*COI* regions. However, the *H. mani* sequences (AY148377 and PQ686670) differ from *H. avenae* (AY148372), with strong support (100% bootstrap value—Figure 4).

The sequence PQ686673, isolated from Coimbra, has 96.48% similarity with EF611116 (*H. schachtii*), 96.37% with AY590283.1 (*H. trifolii*), and 95.85% with LC208690 (*H. betae*)—Table 3. Once again, the sequence of this isolate is in a clade alone when the NCBI tree of *Heterodera* sequences pairwise comparisons are generated, not clustering with *H. schachtii* or *H. betae* (Supplementary Figure S4).

#### 3.2.3. 28S rDNA

**Figure 5:**
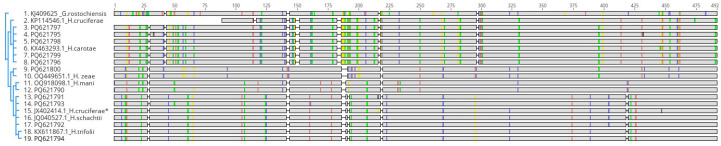
Alignment of 28S rDNA sequences from *Heterodera* species. Rows correspond to individual sequences with GenBank accession and species. Green: conserved nucleotides; yellow: partially conserved; blue/red: species-specific substitutions. Numbers above indicate nucleotide positions. The alignment reveals patterns of conservation and divergence. * Sequence misidentified as *H. cruciferae*, as it should be identified as *H. schachtii*.

PCR amplification with the primers D2A/D3B [33] produced a fragment ranging between 718 bp and 745 bp across all the studied isolates. Of these, 97 bp at the 5′ end and 167 bp at the 3′ end were highly conserved and excluded from the analysis, resulting in a final fragment of 492 bp for the study. This fragment showed high variability (Figure 5).

**Figure 6:**
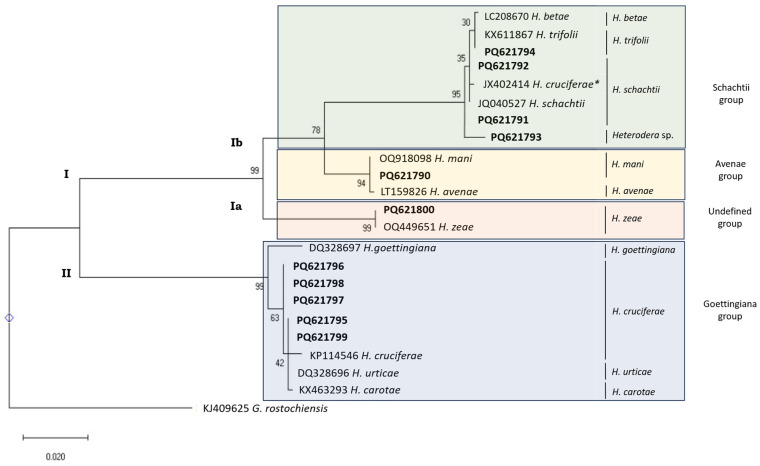
Phylogenetic relationships of *Heterodera* isolates from Portugal (bold) inferred based on the sequence alignment of thefrom 28S rDNA region. The evolutionary history was reconstructed by using the Maximum Likelihood method with the Tamura–Nei model [39] in MEGA X [36]. Bootstrap support (1000 replicates indicated next to branches. Initial trees for the heuristic search were generated via Maximum Parsimony. Rate variation modeled with discrete Gamma (+G, 5 categories, 0.1884), with 0.00% invariable sites (+I). Branch lengths represent substitutions per site. Dataset: 23 sequences, 667 positions. *Globodera rostochiensis* was used as the outgroup taxon. * Sequence misidentified as *H. cruciferae*, as it should be identified as *H. schachtii*.

The phylogenetic relationships of *Heterodera* species based on the 28S rDNA region (Figure 6) show two major clades. In the first clade (I), two sub-clades are formed: Sub-clade Ia (in orange) with *H. zeae* (PQ621800 and OQ449651) sequences, allowing for the identification of the isolate from Santarém/Golegã as *H. zeae.* Sub-clade Ib is divided between the Avenae group (in yellow) and the Schachtii group (in green). Within the Avenae group, *H. mani* (OQ449651 and PQ621790) and *H. avenae* (LT159826) sequences clustered together, not allowing for species identification of the isolate from Castelo Branco (PQ621790). The species identification of this isolate is based on the mt*COI* and ITS-rDNA sequences, which clearly differentiate *H. mani* from *H. avenae*. Within the Schachtii group, *H. trifolii* (KX611867, PQ621794), *H. schachtii* (JQ040527, JX402414, PQ621791, and PQ621792), and *H. betae* (LC208670) sequences clustered together with the unidentified species sequence from the isolate from Coimbra (PQ621793), not allowing for species identification. These results are in accordance with Huston et al. [1], who report that *Heterodera schachtii* and *H. betae* sequences differ from each other by only one bp, while *H. schachtii* and *H. trifolii* sequences differ by two bp in the 28S gene region, making comparison with other genetic markers necessary to identify this species. The species identification of the isolate from Viana do Castelo (PQ621794) as *H. trifolii* is based on the ITS-rDNA (using the primers 18L/ITS4mod) sequences, which clearly differentiate *H. trifolii* from *H. betae and H. schachtii*. The species identification of the isolates from Leiria (PQ621791) and Faro (PQ621792) as *H. schachtii* is based on the mt*COI* and ITS rDNA (using the primers 18L/ITS4mod) sequences, which clearly differentiate *H. schachtii* from *H. betae and H. trifolii*. In clade II, the *Goettingiana* group clade (in blue), two sub-clades are formed: one sub-clade with the *H. goettingiana* (DQ328697) species sequence, and in the second sub-clade, *H. urticae* (DQ328696), *H. carotae* (KX463293), and *H. cruciferae* (KP114546 and PQ621795-PQ621799) species sequences clustered together, not allowing for species identification.

The sequence PQ621793, from Coimbra, has 99.46% similarity with MK895554 (*H. schachtii*) and 99.19% with MW376563 (*H. glycines*) and KX611867 (*H. trifolii*). Once again, the PQ621793 sequence is in a clade alone when the NCBI tree of *Heterodera* sequences is generated using pairwise comparisons (Supplementary Figure S5), not clustering with *H. schachtii*, *H. glycines*, or *H. trifolii*.

Prior to this study, only two sequences of *H. cruciferae* were available for comparison in the NCBI database, based on the 28S rDNA region. The JX402414 sequence, according to the present study and to Huston et al. [1], is misidentified as *H. cruciferae*, as it should have been identified as *H. schachtii*. On the other hand, the KP114546 sequence is well identified as *H. cruciferae*, but it only has 551 bp, while the length expected is higher than 730 bp, mistaking *H. cruciferae* species identification with *H. carotae*, *H. goettingiana*, or *H. urticae*, as can be observed in Figure 6. However, the isolates sequences from Aveiro (PQ621797), Lisbon (PQ621796), Santarém/Salvaterra (PQ621798), and Vila Real (PQ621799), despite their high similarity with *H. carotae* and *H. urticae* sequences (higher than 99%), clustered together with *Heterodera* sp. sequences, in a different clade of *H. carotae* and *H.* u*rticae*, when the NCBI tree of *Heterodera* sequences pairwise comparisons is generated (Supplementary Figure S6). These results are consistent with Subbotin et al. [8], who reported that identical ITS sequences can be found in morphologically clearly distinct *Heterodera* species such as *H. carotae*/*H. cruciferae*. Species identification as *H. cruciferae*, in this case, is based on the mt*COI* sequences, which clearly differentiate *H. cruciferae* from *H. carotae* and *H. goettingiana*, and on ITS rDNA sequences (using the primers 18L/ITS4mod), which clearly differentiate *H. Cruciferae* from *H. carotae*, *H. goettingiana*, and *H. urticae*.

## 4. Discussion

Five valid *Heterodera* species—*Heterodera cruciferae*, *H. mani*, *H. schachtii*, *H. trifolii*, and *H. zeae*—were molecularly identified using mt*COI*, 18S rDNA, ITS, and 28S rDNA markers. To our knowledge, this is the first report of *H. mani* in Portugal, expanding its known distribution within Europe. This has particular significance in light of the EU Plant Health Regulation, which emphasizes early detection and monitoring of harmful organisms to prevent their spread.

Using the same molecular criteria, one potentially undescribed species was also distinguished (represented by sequences PQ462047, PQ686664, PQ621793, and PQ686673) or, at least, a species for which no corresponding sequences of the studied markers are currently available in public databases. This taxon, referred to as *Heterodera* sp. from Coimbra, underscores the challenges in molecular identification due to taxonomic underrepresentation or misidentified sequences in genetic databases.

It is important to emphasize that this study does not aim to formally describe a new *Heterodera* species. Although the molecular results suggest the presence of a distinct lineage, the absence of complementary morphological and morphometric analyses—including cyst cone structure, stylet knobs configuration, J2 tail morphology, and perineal patterns—prevents any definitive taxonomic conclusion. For this reason, we provisionally report this lineage as *Heterodera* sp. and indicate that integrative approaches combining morphological and molecular evidence will be pursued in ongoing and future research to clarify the systematic position of this taxon.

It is important to note that this study does not aim to formally describe a new *Heterodera* species. Although the molecular data suggest the presence of a distinct lineage, the absence of complementary morphological and morphometric analyses—including cyst cone structure, stylet knob configuration, J2 tail morphology, and perineal patterns—prevents any definitive taxonomic conclusion. For this reason, we provisionally report this lineage as *Heterodera* sp. and indicate that integrative approaches combining morphological and molecular evidence will be pursued in ongoing and future research to clarify the systematic position of this taxon.

Such cases underline the importance of integrative taxonomy for providing reliable identifications that can inform quarantine measures and international reporting obligations under EPPO and International Plant Protection Convention frameworks.

Based on the results presented, among the markers tested, the mt*COI* gene amplified with primers JB3/JB5 proved to be the most effective for distinguishing *Heterodera* species. These primers successfully separated *H. trifolii* from *H. schachtii* and *H. cruciferae* from *H. carotae*, supporting their application in routine diagnostic workflows. However, they could not distinguish *H. betae* from *H. trifolii* and *H. cruciferae* from *H. urticae* (differs via ITS-rDNA using the primers 18L/ITS4mod), highlighting the need for additional markers in such cases, where species-level accuracy is essential for compliance with EU phytosanitary measures. This supports the findings of Huston et al. [1], who recommended mt*COI* as the most informative barcode for *Heterodera* species identification, especially when resources are limited.

The results underscore the central role of molecular diagnostics in supporting both scientific and regulatory agendas. For nematode management, reliable species identification enables tailored integrated pest management strategies that minimize yield losses while reducing chemical inputs. For biosecurity, accurate and timely species recognition underpins pest risk analysis, informs decisions on movement restrictions, and strengthens preparedness against invasive nematodes. Overall, given the presence of intraspecific polymorphism and limited reference data, investment in curated sequence databases, harmonized diagnostic protocols, and validated molecular tools is urgently needed. Thus, the development and validation of more robust molecular tools remain essential for accurate diagnostics and taxonomic resolution and reinforce EU biosecurity frameworks and international trade safeguards against cyst nematodes.

For farmers, the ability to correctly identify cyst nematode species has direct and practical consequences. Different *Heterodera* species vary in both host range and damage potential, so precise diagnostics are essential for guiding crop rotation (e.g., avoiding beet or brassica crops in fields with *H. schachtii* or *H. cruciferae*), selecting resistant cultivars, and using nematicides more efficiently. Extension services can then translate molecular diagnoses into region-specific management advice, helping to reduce unnecessary inputs while improving long-term soil health. At a broader level, regulators and diagnostic laboratories provide the foundations for this knowledge transfer by detecting new incursions such as *H. mani*, ensuring that both national surveillance and local farming practices remain aligned with EU phytosanitary goals. Together, these measures help farmers to protect yields while safeguarding worldwide agricultural systems against the spread of damaging cyst nematodes.

## Figures and Tables

**Table 1:** *Heterodera* isolates sequenced in the present study (E-value = 0.0).

Heterodera Species	Locality (District/City)	Collection Code/Year		mtCOI Gene (Expected Frag. Size 447 bp)		18S rDNA Gene (Frag. Size 1730 bp)		ITS rDNA Gene (Frag. Size 1040 bp)		28S rDNA Gene (Frag. Size 780 bp)	
				GenBank Acc. No	bp	GenBank Acc. No.	bp	GenBank Acc. No	bp	GenBank Acc. No	bp
*H*. *cruciferae*	Aveiro/Vagos	1725	2021	PQ462051	390	PQ686668	1700	PQ686676	975	PQ621797	744
*H. cruciferae*	Lisbon/Mafra	1538-1	2020	PQ462050	390	PQ686667	1016	PQ686675	984	PQ621796	741
*H. cruciferae*	Porto/Gondomar	11391	2018	PQ462049	390	PQ686666	860			PQ621795	740
*H. cruciferae*	Santarém/Salvaterra	15732	2018	PQ462052	390	PQ686669	1700			PQ621798	718
*H. cruciferae*	V. Real/Chaves	2140	2020	PQ462053	390					PQ621799	740
*H. mani*	C. Branco/Covilhã	13405	2018	PQ462044	390	PQ686661	1699	PQ686670	978	PQ621790	746
*H. schachtii*	Faro/Loulé	773-4	2020	PQ462046	390	PQ686663	1700	PQ686672	964	PQ621792	744
*H. schachtii*	Leiria/Óbidos	1856-1	2019	PQ462045	390	PQ686662	1700	PQ686671	983	PQ621791	745
*H. trifolii*	V. Castelo/Melgaço	1249-2	2019	PQ462048	390	PQ686665	1700	PQ686674	962	PQ621794	744
*H. zeae*	Santarém/Golegã	1978	2023	PQ462054	390	PV364147	1696	PV491270	568	PQ621800	737
*Heterodera* sp.	Coimbra/Coimbra	1086-1	2019	PQ462047	390	PQ686664	1702	PQ686673	961	PQ621793	744

**Table 2:** Sequences of *Heterodera* spp. available from GenBank used for phylogenetic analysis in the present study.

Heterodera Species	mtCOI Gene GenBank Accession Number		18S rDNA Gene GenBank Accession Number		ITS rDNA Gene GenBank Accession Number		28S rDNA Gene GenBank Accession Number	
*H. avenae*	MG522934	415	KJ636290	1700	AY148372	969	LT159826	801
*H. betae*	MW345308	434	FJ040404 KJ636291	1700 1700	EF611122	1027	LC208670	737
*H. carotae*	MG563235	424	**		MG563237	936	KX463293	749
*H. cruciferae*	MG563234 MW363073	424 424	AY566816	586	MK848393	962	KP114546	551
*H. cruciferae* *							JX402414	747
*H. goettingiana*	MW363088	424	EU669915	1700	AF498374	960	DQ328697	653
*H. mani*	MG523097	415	EU669916	1700	AY148377	968	OQ918098	739
*H. schachtii*	MW345389	434	EU306355	1772	LC208693	985	JQ040527	780
*H. trifolii*	KT163239	391	FJ040402	1699	AY590283	1011	KX611867	754
*H. urticae*	MK093155	868	**		AF274412	962	DQ328696	653
*H. zeae*	OQ449704	391	HQ724313	610	OP692770	1033	OQ449651	715
*G. rostochiensis*	MN095975	443	MZ613180	1740	MN493790	973	KJ409625	784

* Sequence misidentified as *H. cruciferae*, as it should be identified as *H. schachtii*. ** Sequence not available at NCBI database.

**Table 3:** Comparison of isolates with database sequences and species identification.

Locality (District/City)	Marker	Isolate (Study)	Closest GenBank Match	Coverage	% Identity	Notes
***Heterodera Cruciferae* isolates**						
Aveiro/ Vagos	mt*COI*	PQ462051	*H. cruciferae* (MW363073) *H. cruciferae* (MG563234) *H. urticae* (MK093155I) *H. carotae* (MG563235)	100% 100% 87% 100%	100% 99.74% 99.71% 95.38%	Confirmed as *H. cruciferae* by mt*COI*; only one *H. urticae* sequence available for comparison. ITS-rDNA (18L/ITS4mod) and 28S differentiates it from *H. urticae*.
	18S rDNA	PQ686668	*H. cruciferae* (AY566816) *H. goettingiana* (EU669915) *H. carotae* *H. carotae*	34% 100% No data No data	99.83% 99.71% No data No data	Partial 18S rDNA similarity; only one *H. cruciferae* sequence available (586/1700 bp), while *H. goettingiana* sequence is complete, increasing misidentification risk. Identification confirmed by mt*COI* and ITS-rDNA (18L/ITS4mod). This isolate adds a new database contribution.
	ITS	PQ686676	*H. cruciferae* (MK848393) *H. cruciferae* (AF274411) *H. carotae* (MG563237) *H. urticae* (AF274412) *H. goettingiana* (AF498374)	98% 98% 96% 98% 98%	100% 99.90% 99.25% 98.85% 94.69%	Confirmed as *H. cruciferae*; identification supported by mt*COI*, differs from *H. carotae* by mt*COI* and from *H. urticae* by ITS-rDNA (18L/ITS4mod)—99% bootstrap value.
	28S rDNA	PQ621797	*H. cruciferae* (KP114546) *H. urticae* (DQ328696) *H. carotae* (KX463293) *H. goettingiana* (DQ328697)	98% 91% 99% 91%	98.90% 99.85% 99.44% 98.62%	Inconclusive for species identification; only one *H. cruciferae* sequence available (551/730 pb). However, Sequence clusters separately from *H. carotae* and *H. urticae* in NCBI phylogeny. This isolate adds a new database contribution.
Lisbon/ Mafra	mt*COI*	PQ462050	*H. cruciferae* (MW363073) *H. cruciferae* (MG563234) *H. urticae* (MK093155I) *H. carotae* (MG563235)	100% 100% 87% 100%	100% 99.74% 99.71% 95.38%	Confirmed as *H. cruciferae* by mt*COI*; only one *H. urticae* sequence available for comparison. ITS-rDNA (18L/ITS4mod) and 28S differentiates it from *H. urticae*.
	18S rDNA	PQ686667	*H. cruciferae* (AY566816) *H. goettingiana* (EU669915) *H. carotae* *H. carotae*	39% 100% No data No data	99.75% 99.61% No data No data	Partial 18S rDNA similarity; only one *H. cruciferae* sequence available (586/1700 bp), while *H. goettingiana* sequence is complete, increasing misidentification risk. Identification confirmed by mt*COI* and ITS-rDNA (18L/ITS4mod). This isolate adds a new database contribution.
	ITS	PQ686675	*H. cruciferae* (MK848393) *H. cruciferae* (AF274411) *H. carotae* (MG563237) *H. urticae* (AF274412) *H. goettingiana* (AF498374)	95% 98% 95% 98% 98%	100% 99.90% 99.57% 99.17% 95.03%	Confirmed as *H. cruciferae*; identification supported by mt*COI*, differs from *H. carotae* by mt*COI* and from *H. urticae* by ITS-rDNA (18L/ITS4mod)—99% bootstrap value.
	28S rDNA	PQ621796	*H. cruciferae* (KP114546) *H. urticae* (DQ328696) *H. carotae* (KX463293) *H. goettingiana* (DQ328697)	75% 98% 99% 88%	99.10% 99.85% 99.59% 98.62%	Inconclusive for species identification; only one *H. cruciferae* sequence available (551/730 pb). However, sequence clusters separately from *H. carotae* and *H. urticae* in NCBI phylogeny. This isolate adds a new database contribution.
Porto/ Gondomar	mt*COI*	PQ462049	*H. cruciferae* (MW363073) *H. cruciferae* (MG563234) *H. urticae* (MK093155I) *H. carotae* (MG563235)	100% 100% 87% 100%	100% 99.74% 99.71% 95.38%	Confirmed as *H. cruciferae* by mt*COI*; only one *H. urticae* sequence available for comparison. ITS-rDNA (18L/ITS4mod) and 28S differentiates it from *H. urticae*.
	18S rDNA	PQ686666	*H. cruciferae* (AY566816) *H. goettingiana* (EU669915) *H. carotae* *H. carotae*	28% 100% No data No data	98.35% 99.18% No data No data	Partial 18S rDNA similarity; only one *H. cruciferae* sequence available (586/1700 bp), while *H. goettingiana* sequence is complete, increasing misidentification risk. Identification confirmed by mt*COI* and ITS-rDNA (18L/ITS4mod). This isolate adds a new database contribution.
	ITS	–	–	–	–	No amplification
	28S rDNA	PQ621795	*H. cruciferae* (KP114546) *H. urticae* (DQ328696) *H. carotae* (KX463293) *H. goettingiana* (DQ328697)	75% 88% 99% 88%	99.10% 99.69% 99.46% 98.16%	Inconclusive for species identification; only one *H. cruciferae* sequence available (551/730 pb). However, sequence clusters separately from *H. carotae* and *H. urticae* in NCBI phylogeny. This isolate adds a new database contribution.
Santarém/ Salvaterra	mt*COI*	PQ462052	*H. cruciferae* (MW363073) *H. cruciferae* (MG563234) *H. urticae* (MK093155I) *H. carotae* (MG563235)	100% 100% 87% 100%	100% 99.74% 99.71% 95.38%	Confirmed as *H. cruciferae* by mt*COI*; only one *H. urticae* sequence available for comparison. ITS-rDNA (18L/ITS4mod) and 28S differentiates it from *H. urticae*.
	18S rDNA	PQ686669	*H. cruciferae* (AY566816) *H. goettingiana* (EU669915) *H. carotae* *H. carotae*	34% 100% No data No data	99.83% 99.71% No data No data	Partial 18S rDNA similarity; only one *H. cruciferae* sequence available (586/1700 bp), while *H. goettingiana* sequence is complete, increasing misidentification risk. Identification confirmed by mt*COI* and ITS-rDNA (18L/ITS4mod). This isolate adds a new database contribution.
	ITS	–	–	–	–	No amplification
	28S rDNA	PQ621798	*H. cruciferae* (KP114546) *H. urticae* (DQ328696) *H. carotae* (KX463293) *H. goettingiana* (DQ328697)	75% 91% 99% 91%	98.9% 99.85% 99.44% 98.62%	Inconclusive for species identification; only one *H. cruciferae* sequence available (551/730 pb). However, sequence clusters separately from *H. carotae* and *H. urticae* in NCBI phylogeny. This isolate adds a new database contribution.
V. Real/ Chaves	mt*COI*	PQ462053	*H. cruciferae* (MW363073) *H. cruciferae* (MG563234) *H. urticae* (MK093155I) *H. carotae* (MG563235)	100% 100% 87% 100%	99.74% 100% 100% 95.90%	Confirmed as *H. cruciferae* by mt*COI*; only one *H. urticae* sequence available for comparison. ITS-rDNA (18L/ITS4mod) and 28S differentiates it from *H. urticae*.
	18S rDNA	–	–	–	–	No amplification
	ITS	–	–	–	–	No amplification
	28S rDNA	PQ621799	*H. cruciferae* (KP114546) *H. urticae* (DQ328696) *H. carotae* (KX463293) *H. goettingiana* (DQ328697)	75% 88% 99% 88%	99.28% 99.58% 99.86% 98.32%	Inconclusive for species identification; only one *H. cruciferae* sequence available (551/730 pb). However, sequence clusters separately from *H. carotae* and *H. urticae* in NCBI phylogeny. This isolate adds a new database contribution.
***Heterodera mani* isolates**						
C. Branco/ Covilhã	mt*COI*	PQ462044	*H. mani* (MG523097) *H. avenae* (MG522934)	100% 98%	100% 90.86%	Confirmed as *H. mani* by mt*COI*.
	18S rDNA	PQ686661	*H. mani* (EU669916) *H. aveane* (KJ636290)	100% 100%	99.94% 99.88%	Only one *H. mani* sequence is currently available for comparison. Species identification is based on mt*COI* and ITS-rDNA. This isolate provides a new contribution to the database.
	ITS	PQ686670	*H. mani* (AY148377) *H. avenae* (AY148372)	95% 99%	99.69% 99.28%	Species identification is based on mt*COI.* The *H. mani* sequences differ from *H. avenae*, with strong support (100% bootstrap value).
	28S rDNA	PQ621790	*H. mani* (OQ918098) *H. avenae* (LT159826)	99% 100%	99.86% 99.87%	Species identification is based on mt*COI* and ITS-rDNA.
***Heterodera schachtii* isolates**						
Faro/ Loulé	mt*COI*	PQ462046	*H. schachtii* (MW345380) *H. schachtii* (MW345389)	100% 100%	100% 100%	Confirmed as *H. schachtiii* by mt*COI*.
	18S rDNA	PQ686663	*H. schachtii* (EU306355) *H. schachtii* (KJ636284) *H. trifolii* (FJ040402) *H. trifolii* (KJ636287) *H. betae* (KJ636291) *H. betae* (FJ040404)	100% 100% 100% 100% 100% 100%	99.94% 99.88% 99.82% 99.76% 99.94% 99.94%	Inconclusive for species identification; species identification is based on mt*COI* and ITS-rDNA (18L/ITS4mod), which clearly separate *H. schachtii* from both *H. betae* and *H. trifolii*.
	ITS	PQ686672	*H. schachtii* (LC208693) *H. trifolii* (AY590283) *H. betae* (EF611122)	100% 100% 100%	99.9% 98.96% 98.45%	Confirmed as *H. schachtii*; ITS-rDNA is highly variable, enabling species-level identification with strong support (99% bootstrap).
	28S rDNA	PQ621792	*H. schachtii* (JQ040527) *H. schachtii* (MK895555) *H. trifolii* (KX611867)	100% 100% 100%	100% 100% 99.87%	28S-rDNA has limited resolution, as *H. schachtii* differs from *H. betae* by 1 bp and from *H. trifolii* by 2 bp. Species identification relies on mt*COI* and ITS-rDNA (18L/ITS4mod), which clearly separate *H. schachtii* from both *H. betae* and *H. trifolii*.
Leiria/ Óbidos	mt*COI*	PQ462045	*H. schachtii* (MW345380) *H. schachtii* (MW345389)	100% 100%	99.74% 99.74%	Confirmed as *H. schachtii* by mt*COI*.
	18S rDNA	PQ686662	*H. schachtii* (EU306355) *H. schachtii* (KJ636284) *H. trifolii* (FJ040402) *H. trifolii* (KJ636287) *H. betae* (KJ636291) *H. betae* (FJ040404)	100% 100% 100% 100% 100% 100%	100% 99.94% 99.88% 99.82% 99.94% 100%	Inconclusive for species identification; species identification is based on mt*COI* and ITS-rDNA (18L/ITS4mod), which clearly separate *H. schachtii* from both *H. betae* and *H. trifolii*.
	ITS	PQ686671	*H. schachtii* (LC208693) *H. trifolii* (AY590283) *H. betae* (EF611122)	99% 99% 99%	99.48% 98.76% 98.25%	Confirmed as *H. schachtii*; ITS-rDNA is highly variable, enabling species-level identification with strong support (99% bootstrap). The sequences in this gene region are highly variable, allowing for the identification of isolates as *H. schachtii*, highly supported by a 99% bootstrap value
	28S rDNA	PQ621791	*H. schachtii* (JQ040527) *H. schachtii* (MK895555) *H. trifolii* (KX611867)	100% 100% 100%	99.60% 99.60% 99.46%	28S-rDNA has limited resolution, as *H. schachtii* differs from *H. betae* by 1 bp and from *H. trifolii* by 2 bp. Species identification relies on mt*COI* and ITS-rDNA (18L/ITS4mod), which clearly separate *H. schachtii* from both *H. betae* and *H. trifolii*.
***Heterodera trifolii* isolates**						
V. Castelo/ Melgaço	mt*COI*	PQ462048	*H. trifolii* (KT163239) *H. trifolii* (MK621902) *H. betae* (MW345308) *H. betae* (LC208706)	100% 100% 100% 100%	99.74% 99.74% 99.74% 99.74%	mt*COI* ambiguous (1 bp difference, low resolution); species identification confirmed by ITS-rDNA (18L/ITS4mod).
	18S rDNA	PQ686665	*H. schachtii* (EU306355) *H. betae* (FJ040404) *H. betae* (KJ636291) *H. trifolii* (FJ040402) *H. trifolii* (KJ636287)	100% 100% 100% 100% 100%	100% 100% 99.94% 99.88% 99.82%	18S rDNA ambiguous (1–2 bp differences; ITS regions shared among species); identification confirmed by ITS-rDNA (18L/ITS4mod)
	ITS	PQ686674	*H. trifolii* (AY590283) *H. betae* (EF611122) *H. schachtii* (LC208693)	100% 100% 100%	100% 99.27% 99.06%	ITS-rDNA confirms *H. trifolii*; distinguished from *H. betae* by 7 nucleotide differences.
	28S rDNA	PQ621794	*H. trifolii* (KX611867) *H. betae* (LC208670) *H. schachtii* (MK895555)	100% 99% 100%	100% 99.86% 99.87%	28S-rDNA shows low resolution (*H. schachtii* vs. *H. betae* differ by 1 bp; vs. *H. trifolii* by 2 bp); identification confirmed by ITS-rDNA (18L/ITS4mod)
***Heterodera zeae* isolates**						
Santarém/ Golegã	mt*COI*	PQ462054	*H. zeae* (OQ449704)	93%	100%	Confirmed species
	18S rDNA	PV364147	*H. zeae* (HQ724313) *H. mani* (EU669916) *H. avenae* (KJ636290)	36% 100% 100%	99.84% 98.53% 98.47%	Species identification is based on mt*COI* and 28S.
	ITS	PV491270	*H. zeae* (OP692769) *H. zeae* (OP692770)	100% 100%	99.12% 98.95%	Species identification is based on mt*COI* and 28S.
	28S rDNA	PQ621800	*H. zeae* (OQ449651) *H. zeae* (GU145612)	96% 100%	100% 99.86%	Confirmed species
***Heterodera* sp. isolates**						
Coimbra/ Coimbra	mt*COI*	PQ462047	*H. sonchophila* (MW345341) *H. glycines* (LC208712) *H. medicaginis* (MW345339)	99% 99% 99%	93.25% 92.99% 92.99%	Forms a distinct sub-clade; identification ambiguous, possibly representing an undescribed species.
	18S rDNA	PQ686664	*H. betae* (FJ040404) *H. schachtii* (EU306355) *H. schachtii* (KJ636284) *H. betae* (KJ934138) *H. hordecalis* (FJ040405)	100% 100% 100% 100% 100%	99.72% 99.72% 99.65% 99.65% 99.65%	Forms a distinct sub-clade; identification ambiguous, possibly representing an undescribed species.
	ITS	PQ686673	*H. schachtii* (EF611116) *H. trifolii* (AY590283) *H. betae* (LC208690)	100% 100% 100%	96.48% 96.37% 95.85%	Unique sub-clade; ambiguous; potentially undescribed species
	28S rDNA	PQ621793	*H. schachtii* (MK895554) *H. schachtii* (JQ040527) *H. glycines* (KP324916) *H. trifolii* (KX611876)	100% 100% 100% 100%	99.46% 99.33% 99.19% 99.19%	Unique sub-clade; ambiguous; potentially undescribed species

## Data Availability

The original contributions presented in this study are included in the article/Supplementary Materials. Further inquiries can be directed to the corresponding author.

## Supplementary Materials

The following supporting information can be downloaded at: https://www.mdpi.com/article/10.3390/pathogens14101052/s1, Figure S1: Pairwise comparisons mt*COI* tree of *Heterodera* sequences, with PQ462047 sequence; Figure S2: Pairwise comparisons 18S rDNA tree of *Heterodera* sequences, with PQ686666–PQ686669 sequences; Figure S3: Pairwise comparisons 18S rDNA tree of *Heterodera* sequences, with PQ686664 sequence; Figure S4: Pairwise comparisons ITS rDNA tree of *Heterodera* sequences, with PQ686673 sequence; Figure S5: Pairwise comparisons 28S rDNA tree of *Heterodera* sequences, with PQ621793 sequence; Figure S6: Pairwise comparisons 28S rDNA tree of *Heterodera* sequences, with PQ621795–PQ621799 sequences.
