# Supplementary figures and images for: Multi-Marker Approach for the Identification of Different Heterodera Species (Nematoda: Heteroderidae)
Source: Pathogens. 2025 Oct 18;14(10):1052. doi: 10.3390/pathogens14101052 (PMC12566900; doi:10.3390/pathogens14101052)

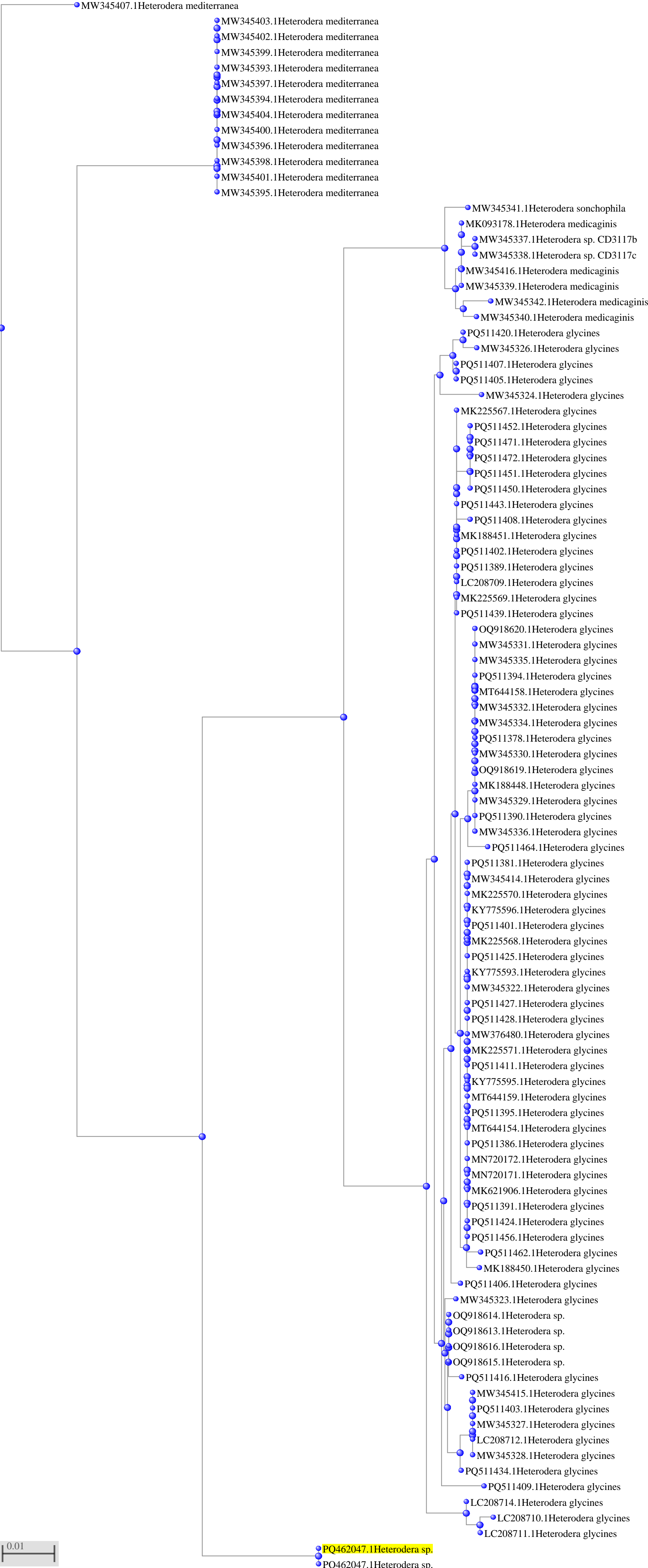

Supplement: Supplementary file 1 [file pathogens-14-01052-s001.zip › Supplementary_Figure_S1.pdf]

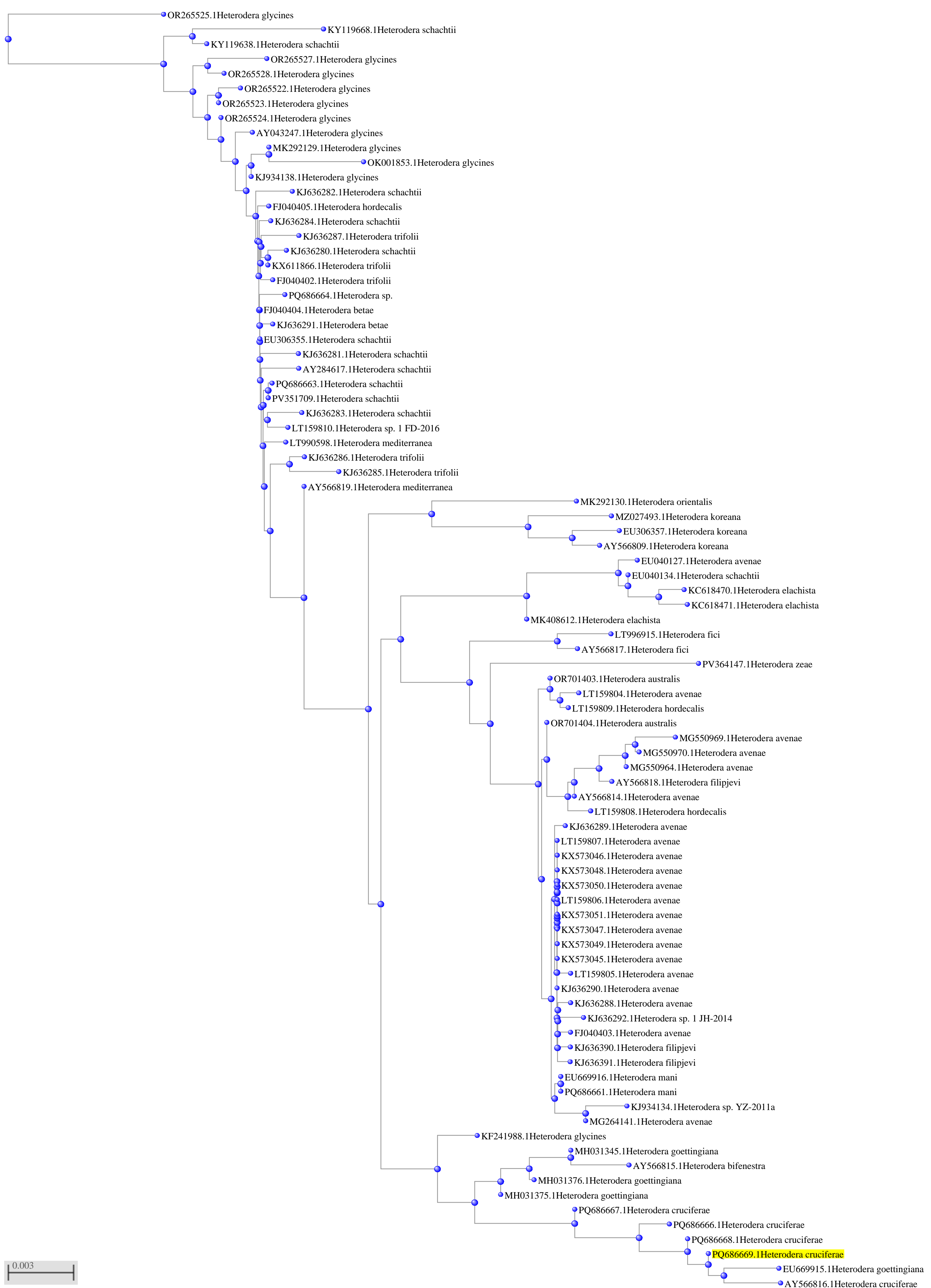

Supplement: Supplementary file 1 [file pathogens-14-01052-s001.zip › Supplementary_Figure_S2.pdf]

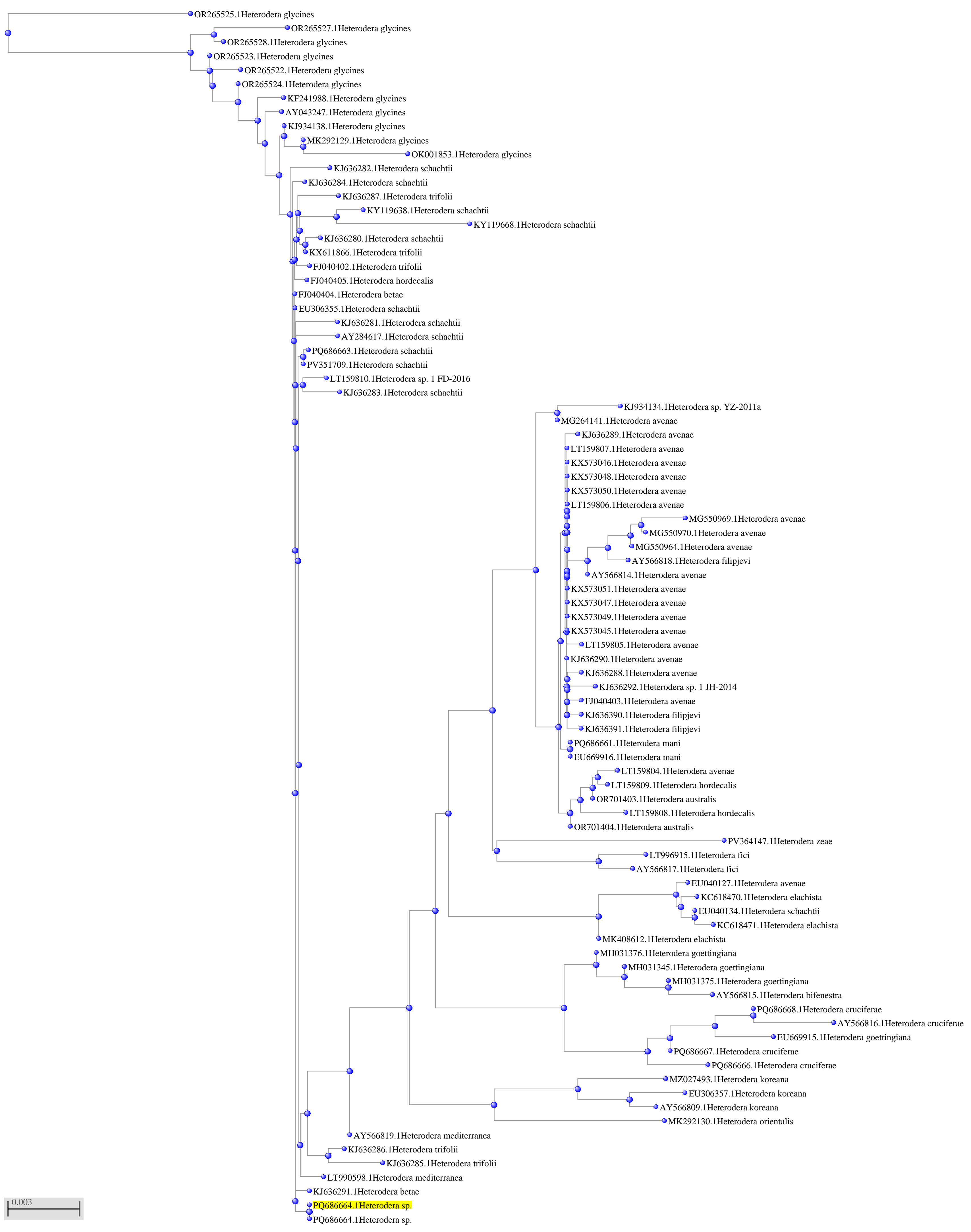

Supplement: Supplementary file 1 [file pathogens-14-01052-s001.zip › Supplementary_Figure_S3.pdf]

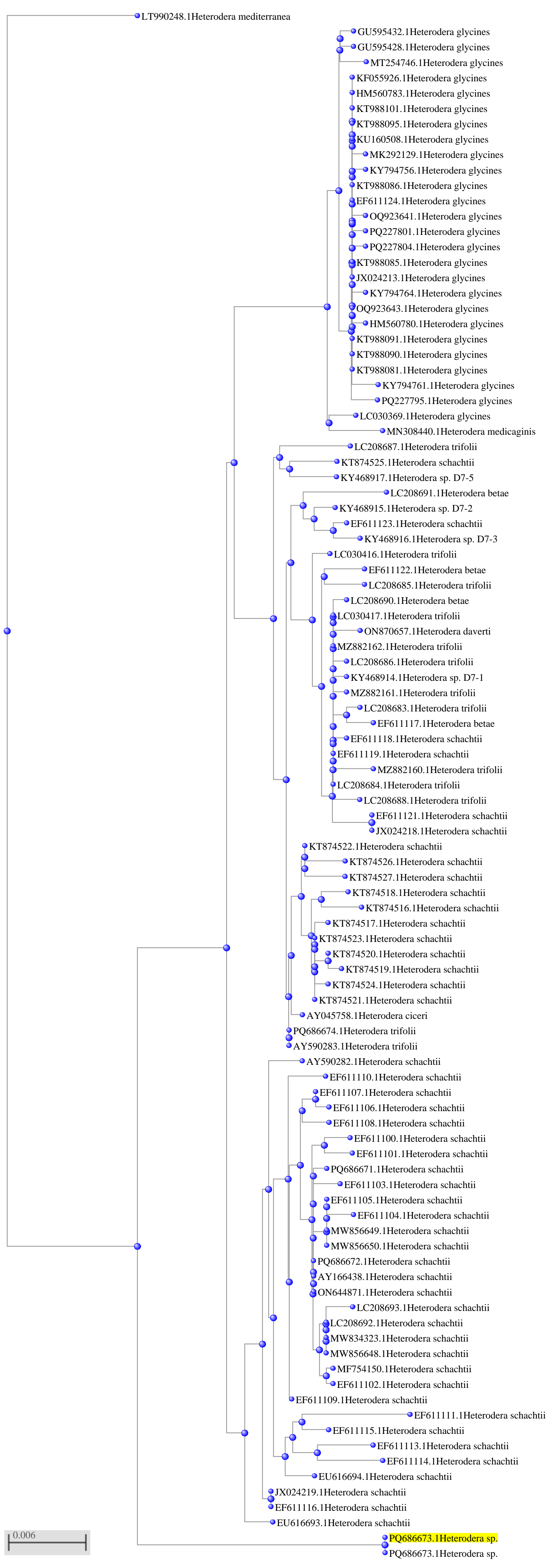

Supplement: Supplementary file 1 [file pathogens-14-01052-s001.zip › Supplementary_Figure_S4.pdf]

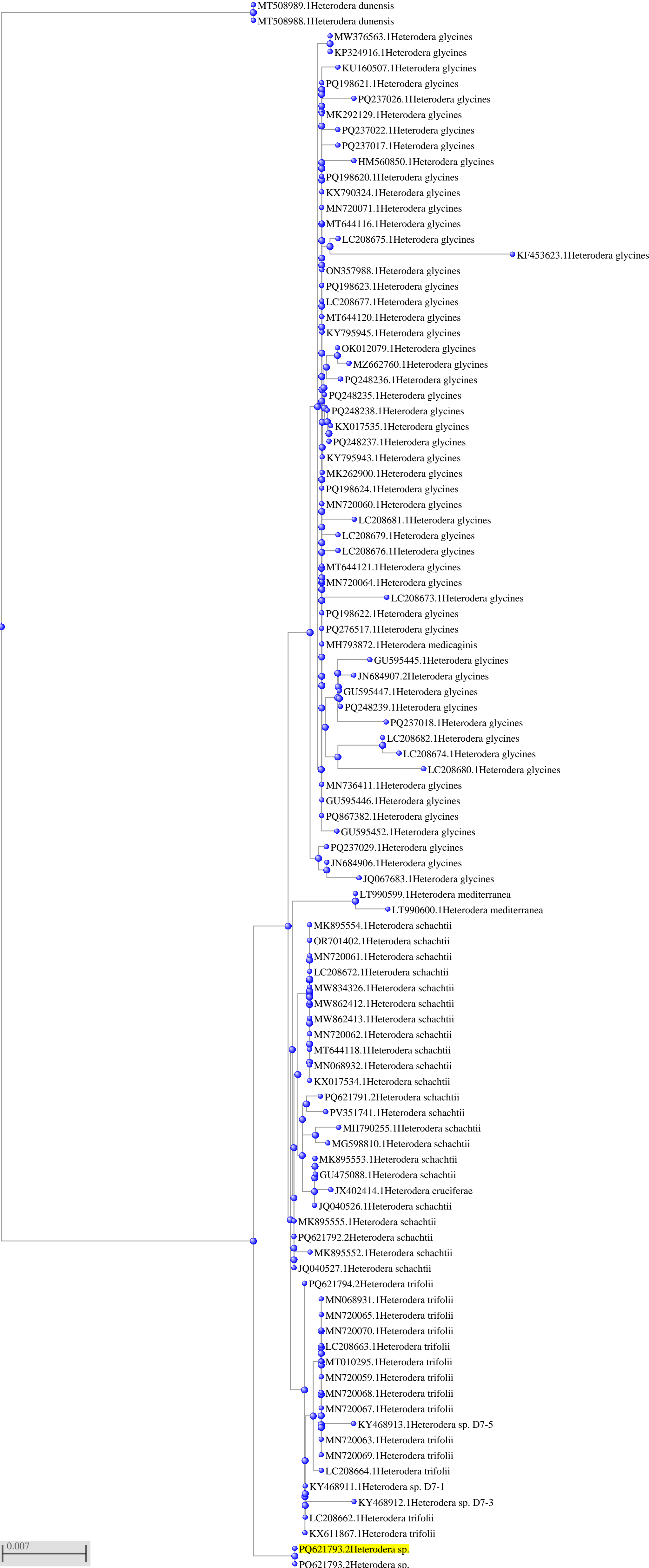

Supplement: Supplementary file 1 [file pathogens-14-01052-s001.zip › Supplementary_Figure_S5.pdf]

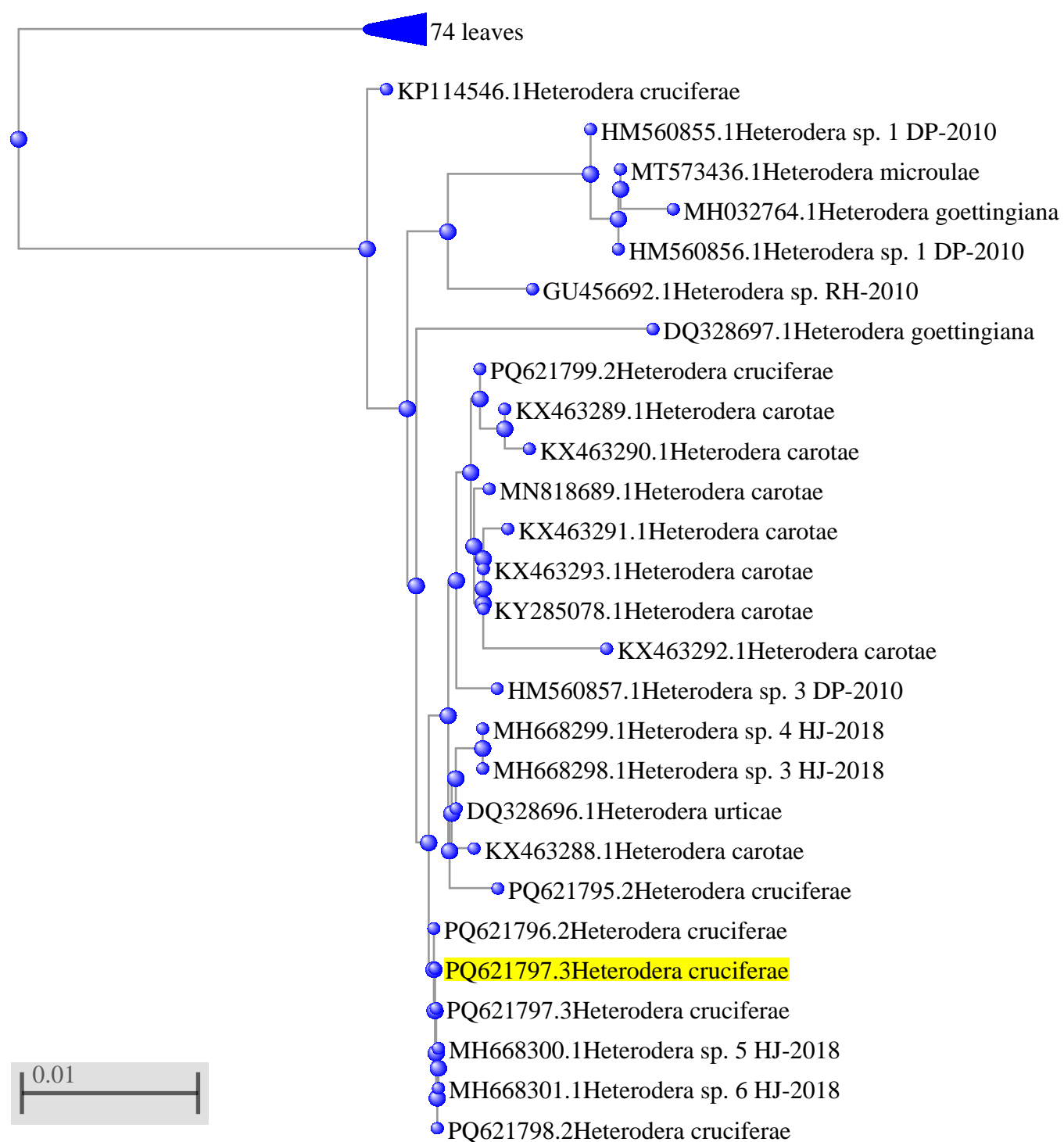

Supplement: Supplementary file 1 [file pathogens-14-01052-s001.zip › Supplementary_Figure_S6.pdf]
